# Myopericarditis in a Patient With Cryoglobulinemic Kidney Disease: A Case Report

**DOI:** 10.7759/cureus.75550

**Published:** 2024-12-11

**Authors:** Soufiane Touiti, Salima Serroukh, Aatif Benyass, Tarik Bouattar

**Affiliations:** 1 Cardiology, Ibn Sina Hospital, Rabat, Rabat, MAR; 2 Cardiology, Mohammed V Military Training Hospital, Rabat, MAR; 3 Nephrology, Ibn Sina Hospital, Rabat, Rabat, MAR; 4 Cardiology, Mohammed V Military Instruction Hospital of Rabat, Mohammed V University, Rabat, MAR

**Keywords:** cryoglobulinemia, heart failure, myocarditis, pericarditis, vasculitis

## Abstract

Cryoglobulinemic vasculitis is a rare small-vessel vasculitis leading to multi-organ dysfunction, often associated with chronic infections like hepatitis C virus (HCV), and autoimmune disorders. Most cases involve mixed monoclonal or polyclonal immunoglobulins, presenting symptoms such as purpura, arthralgias, and weakness. Severe organ involvement, particularly cardiac, is rare but potentially life-threatening.

We report the case of a 48-year-old woman without prior medical history who presented with acute dyspnea, generalized petechial purpura, and signs of global heart failure. Imaging and laboratory findings indicated cardiomegaly, pericardial effusion, and significant nephrotic syndrome with renal failure. The diagnosis of cryoglobulinaemia was confirmed through histology and serology, showing monoclonal IgM with kappa hypergammaglobulinaemia and complement consumption. Treatment included various immunosuppressants, corticosteroids, and rituximab combined with renal replacement therapy. Following the initiation of treatment and proper management of heart failure, the patient's condition significantly improved.

Cardiac involvement in cryoglobulinemic vasculitis, though rare, can lead to severe heart failure. This often involves necrotizing vasculitis of the coronary arteries or systemic inflammation damaging the cardiac muscle, as observed here. Cardiac manifestations with immunosuppressive therapy are reversible despite a poor long-term prognosis for patients with cardiac lesions.

In conclusion, cryoglobulinemic vasculitis has a grim prognosis due to its multi-organ impact and the severity of the lesions. Early and aggressive treatment is essential to manage life-threatening acute presentations, even before confirming the diagnosis biologically or histologically.

## Introduction

Cryoglobulinemic vasculitis is a rare and complex disorder characterized by the presence of cryoglobulins-abnormal proteins that precipitate at low temperatures, leading to inflammation of small and medium-sized blood vessels (vasculitis). This condition primarily affects individuals with underlying chronic infections or autoimmune diseases, with an estimated prevalence of 2-3 cases per 100,000 individuals in the general population. However, it is significantly more common in specific high-risk groups, particularly among those infected with hepatitis C virus (HCV), where the prevalence can be as high as 30% [1].

Cryoglobulinemic vasculitis is classified into three main types based on the underlying cause of the cryoglobulins [2]: type I (monoclonal) is associated with plasma cell disorders such as multiple myeloma, and this type is characterized by a single type of abnormal immunoglobulin; type II (mixed monoclonal and polyclonal) is the most common form and is often linked to chronic infections like HCV, presenting with both monoclonal and polyclonal immunoglobulins; type III (polyclonal) is typically associated with autoimmune diseases such as systemic lupus erythematosus and rheumatoid arthritis and involves multiple types of immunoglobulins.

Clinically, cryoglobulinemic vasculitis typically presents with a triad of symptoms: purpura, arthralgia, and weakness. Other common manifestations include fatigue, renal impairment, neuropathy, and abdominal pain, as the disease can affect various organs, including the skin, kidneys, nerves, and gastrointestinal tract. Understanding the pathophysiology and clinical manifestations of cryoglobulinemic vasculitis is crucial for early recognition and management, as delayed treatment can lead to significant morbidity and potentially fatal outcomes.

## Case presentation

A 48-year-old woman with no previous history was admitted to the emergency department with dyspnea at rest. The symptoms appeared one month prior to admission with the rapidly progressive onset of New York Heart Association (NYHA) stage II dyspnea associated with oedema of the lower limbs extending to the thighs and arthralgia. After one week, the condition worsened, and the dyspnea became NYHA stage III with orthopnea and asthenia with anorexia.

Physical examinations at admission found a conscious patient, with a respiratory rate of 33, heart rate at 127 bpm, 92% pulsed oxygen saturation in ambient air, apyretic and the full-day urine volume was 200 mL. Her blood pressure was 130/65 mmHg. The patient showed signs of heart failure such as oedema in the lower limbs, crackles reaching pulmonary apices, and hepatomegaly. Heart sounds were dampened and the pulse was weakly felt on both sides. Skin examination revealed generalized petechial purpura. The remainder of the examination was unremarkable.

**Figure 1:**
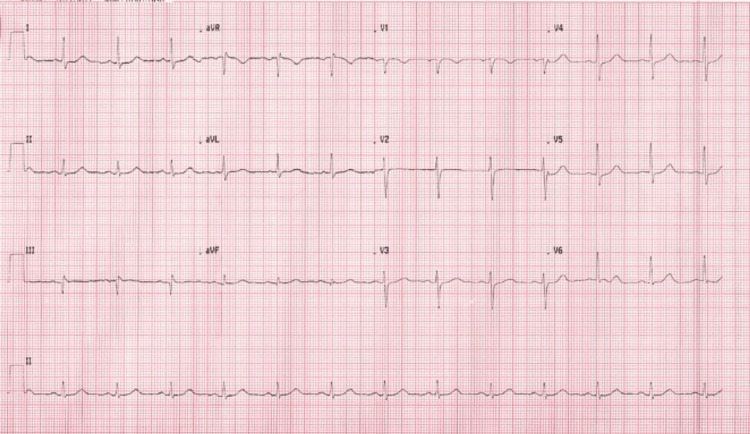
ECG with no abnormalities.

**Figure 2:**
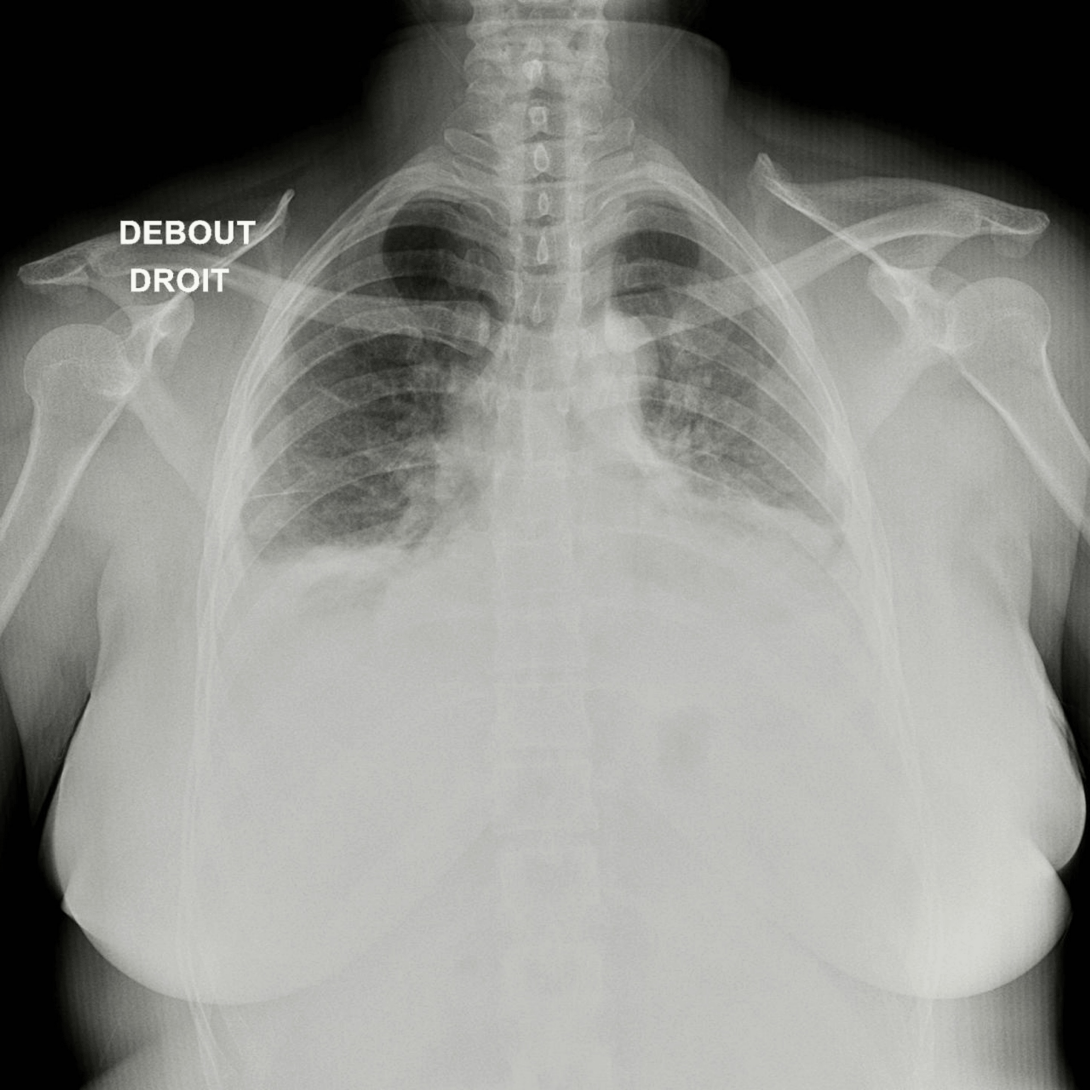
The chest X-ray showed an enlarged heart size with a classical batwing peri-hilum pattern sign and a pleural effusion.

On the first day of admission, the ECG (Figure 1) showed a regular sinus rhythm with a heart rate of 78 bpm and no evidence of ST-T segment repolarization abnormalities. The chest X-ray (Figure 2) showed an enlarged heart size with a classical batwing peri-hilum pattern sign and the cardiac ultrasound showed a moderate circumferential pericardial effusion (11 mm in front of and behind the right ventricle and 12 mm behind the right atrium) with a right atrial collapse and left ventricular dilatation and global hypokinesia with moderate dysfunction (left ventricular ejection fraction (LVEF) of 35%).

**Figure 3:**
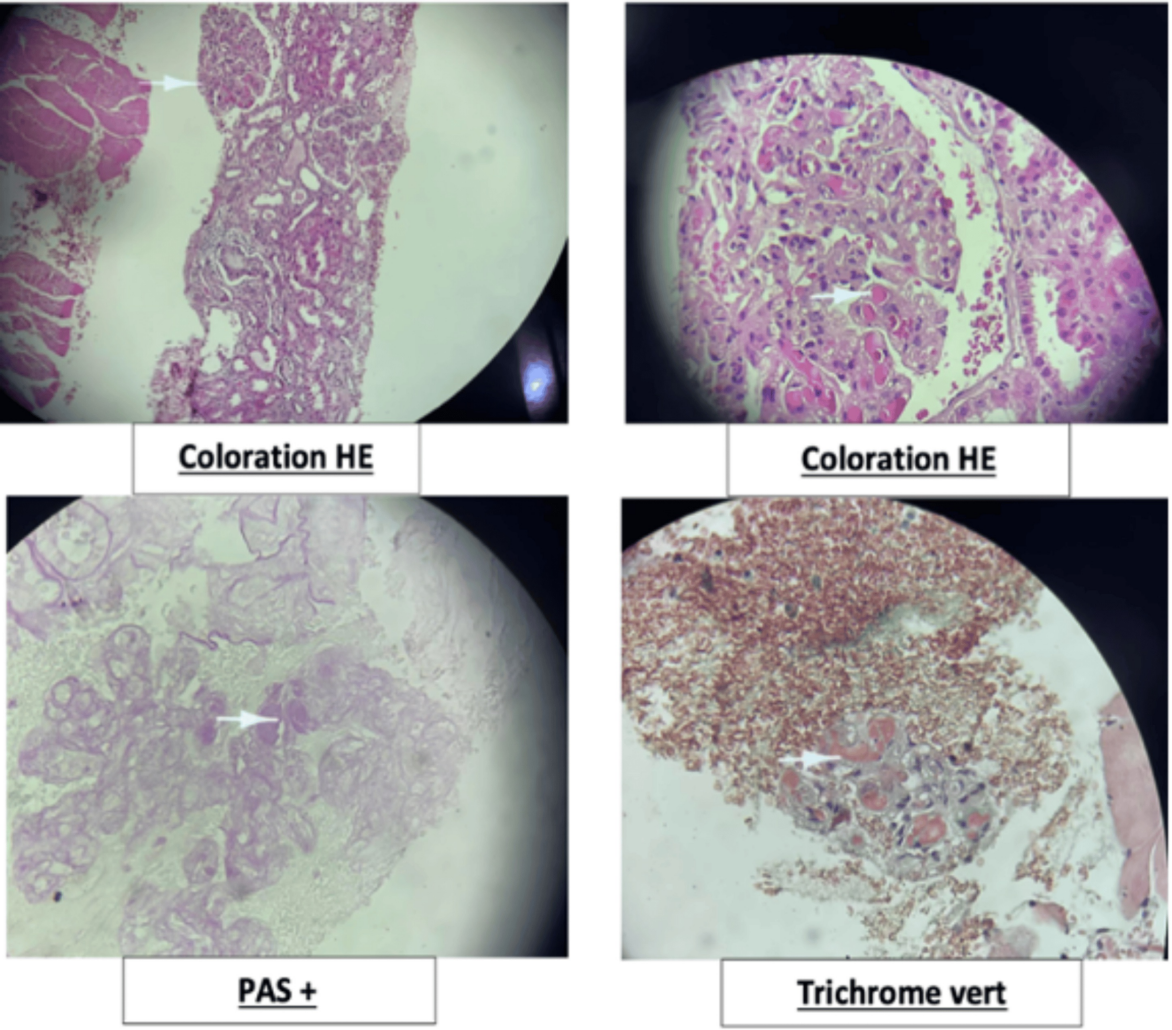
Histological aspect in favour of cryoglobulinaemia on kidney biopsy. HE: hematoxylin and eosin; PAS: periodic acid-Schiff

Laboratory tests revealed normocytic normochromic anaemia at 6.7 g/dL (12-16 g/dL), leukopenia and lymphopenia. In terms of renal function, blood tests revealed an impure nephrotic syndrome with high glomerular proteinuria (10.13 g/day) associated with acute renal failure classified as glomerular filtration rate (GFR) 4. Additionally, active urinary sediment was observed, with hematuria of 103/mm³ and leukocyturia of 101/mm³. Table 1 provides additional biological information. The autoimmune analysis returned negative for antinuclear antibodies (ANA), anti-neutrophil cytoplasmic antibodies (ANCAs), anti-DNA, anti-glomerular basement-membrane (anti-GBM), and anti-cardiolipin antibodies. In addition, there was consumption of complement fractions C3 and C4 and monoclonal IgM kappa hypergammaglobulinaemia. Further tests confirmed the presence of cryoglobulinaemia, but the result was positive several days after the initial blood test. Other tests including tumour markers, infectious diseases (serology and polymerase chain reaction (PCR) for human immunodeficiency virus and hepatitis B and C viruses, and repeated blood cultures) and haematology (myelogram, bone marrow biopsy and salivary gland biopsy) were negative. A kidney biopsy was conducted to confirm the diagnosis (Figure 3).

**Table 1:** Details of the biological characteristics. HIV: human immunodeficiency virus; HCV: hepatitis C virus; HBV: hepatitis B virus; IgG: immunoglobulin G; IgA: immunoglobulin A; IgM: immunoglobulin M

Characteristics	Patient Value	Normal Value
Blood Cell Count		
Hemoglobin (g/dL)	6.9	12-16
Mean corpuscular volume (MCV) (fL)	93	80-100
Mean corpuscular haemoglobin concentration (MCHC) (g/dL)	32	32-36
White blood cell (/mm^3^)	5970	4000-10000
Platelet (/mm^3^)	143000	150000-450000
Renal Function		
Creatinin (mg/L)	28	7.2-12.5
Autoimmune Analysis		
Cryoglobin (mg/L)	Positive	
Anti-nuclear antibody (ANA)	Negative	
Anti-neutrophil cytoplasmic antibodies (ANCAs)	Negative	
Anti-DNA	Negative	
Anti-glomerular basement-membrane (anti-GBM)	Negative	
Anti-cardiolipin	Negative	
C3 (g/L)	0.36	0.82-1.85
C4 (g/L)	0.03	0.15-0.53
Infectious Markers		
Cytobacteriological Examination of Urine (CBEU) (/mm^3^)	Leukocyturia: 73	
	Hematuria: 192	
	Culture: Negative	
Serological markers of HIV	Negative	
Serological markers of HCV	Negative	
Serological markers of HBV	Negative	
Serum Protein Electrophoresis		
Albumin (g/L)	21	35-52
Alpha-1-globulin (g/L)	4.13	
Alpha-2-globulin (g/L)	4.8	
Beta-1-globulin (g/L)	2.62	
Beta-2-globulin (g/L)	1.12	
Gamma globulin (g/L)	6.16	
Immunofixation	Ig monoclonal isotype with IgM kappa	
IgG (g/L)	4.38	5.52-16.3
IgA (g/L)	1.12	
IgM (g/L)	3.79	0.33-2.93

**Figure 4:**
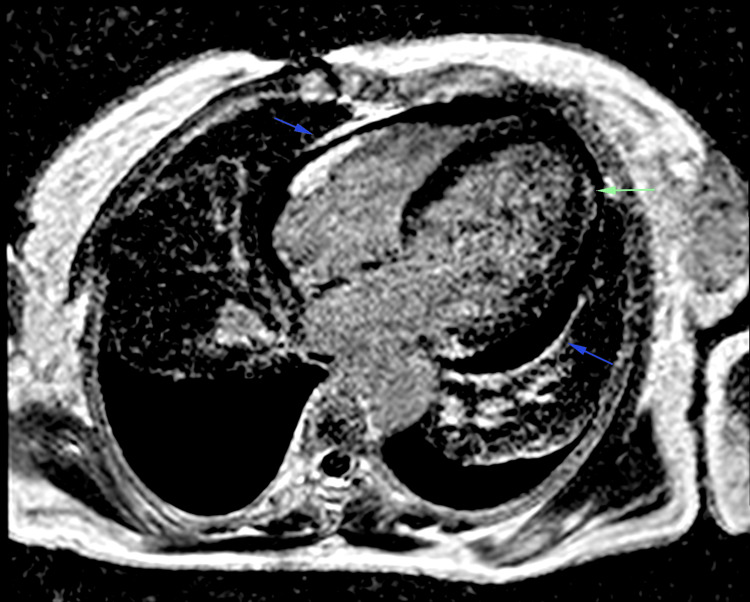
STIR MRI sequence in a four-chamber view showing late enhancement in the subepicardial medio-lateral walls (indicated by green arrow) and pericardial leaflet (indicated by blue arrows). STIR MRI: short-tau inversion recovery magnetic resonance imaging

**Figure 5:**
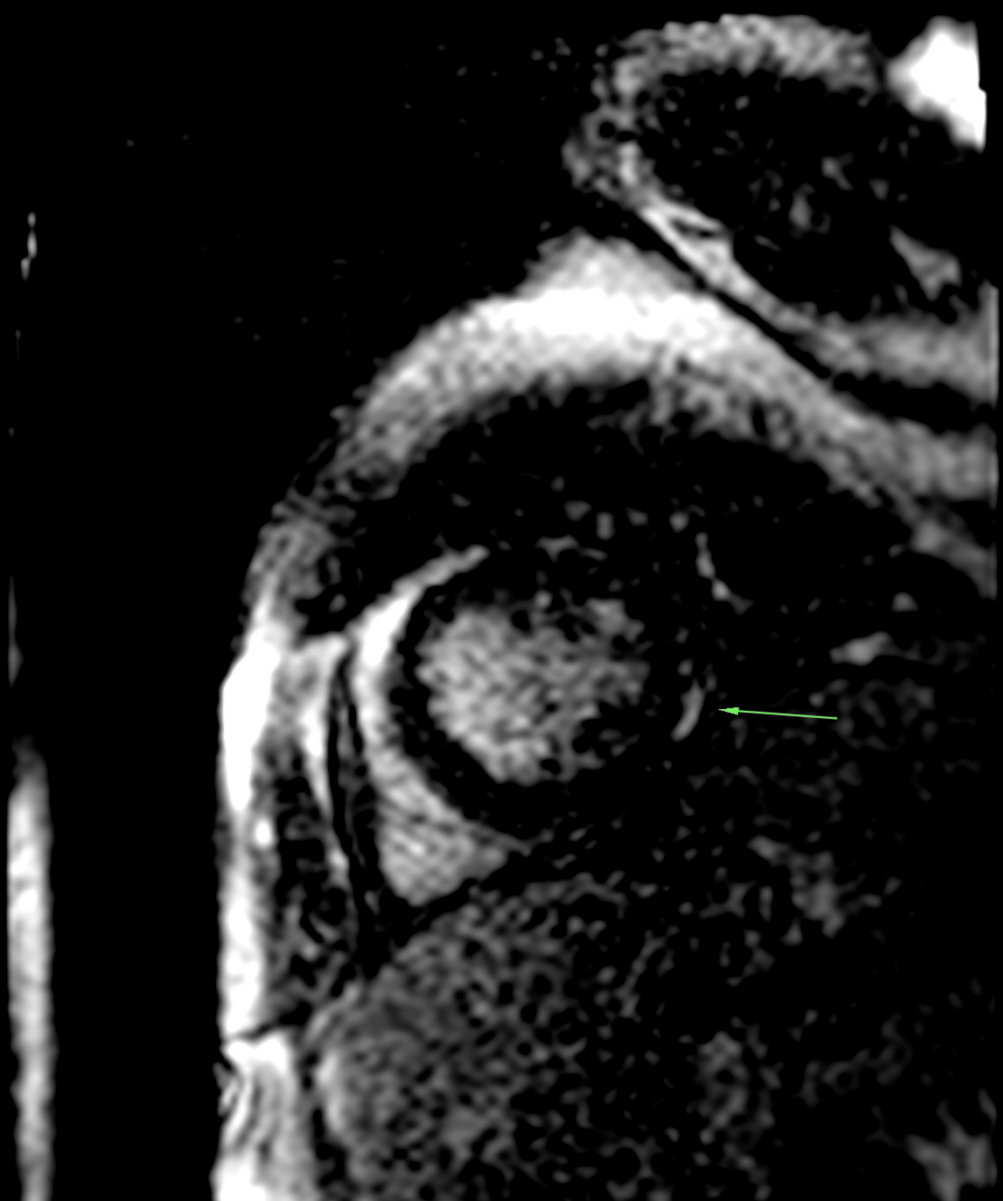
STIR MRI sequence in a short-axis view showing late subepicardial enhancement of the lateral wall of the left ventricle (LV) suggesting myocardial oedema in these areas (indicated by green arrow). STIR MRI: short-tau inversion recovery magnetic resonance imaging

The patient was at first treated with systemic corticosteroids (daily bolus of 500 mg of methylprednisolone for three successive days) with sessions of extra-renal purification. Troponin assays were negative, and together with echocardiographic findings, effectively ruled out myocardial ischemia. Further magnetic resonance imaging (MRI) (Figures 4-5 and Video 1) examination revealed dilated cardiomyopathy with reduced ejection fraction of 40%, and late gadolinium enhancement on both ventricular lateral walls, associated with moderate pericardial and pleural effusion.

**Video 1:** Gradient-echo MRI sequence (white blood) in four-chamber view, in cine mode, demonstrating dilated cardiomyopathy with reduced ejection fraction (40%) with global hypokinesia associated with moderate pericardial and pleural effusion. Dimensional Measurements: Left ventricular internal end-diastolic diameter: 63 mm Left ventricular internal end-systolic diameter: 53 mm Right ventricular internal end-diastolic diameter: 35 mm Right ventricular internal end-systolic diameter: 21 mm

In this context, the diagnosis of essential cryoglobulinaemia with renal and cardiac failure was established. After haemodynamic and clinical stabilisation, therapy based on rituximab and treatment of heart failure adapted to clearance was started. Evolution was favourable and the patient was discharged on optimal medical treatment. She was seen after one month in consultation with a significant improvement in her GFR (34 mL/min) and cardiac function (LVEF 50%).

## Discussion

The broader context of cardiovascular disease (CVD) cannot be fully understood without considering the significant impact of socioeconomic and racial factors on outcomes and prevalence. Individuals from lower socioeconomic backgrounds and racial minorities face higher risks for CVD due to a complex interaction of social, environmental, and healthcare factors [3]. In the case of cryoglobulinemic vasculitis with cardiac involvement, these socioeconomic and racial disparities can have a direct impact on disease progression and survival [4].Limited access to diagnostic tools (e.g., cardiac imaging, cryoglobulin assays) and advanced therapies (e.g., rituximab) may result in delayed or inadequate treatment for patients from disadvantaged backgrounds. This underscores the importance of addressing healthcare inequities to improve outcomes, particularly in vulnerable populations [5].

Microvascular damage in cryoglobulinemic vasculitis, especially when it causes heart failure, is not well documented in the literature [1,2,6]. The presence of late gadolinium enhancement on MRI images of patients with both cryoglobulinaemia and heart failure would lean toward a myocardial microvascular ischemic mechanism, though histopathological examinations are needed to strengthen this theory [6]. Although rare, cardiac involvement occurs in 4-6% of patients and is not related exclusively to HCV infection [7]. It generally appears in the presence of kidney, skin and gastrointestinal attacks, and can lead to severe heart failure. This failure is due to necrotising vasculitis lesions generally affecting the coronary microcirculation and sparing the main arteries, manifesting as chest discomfort or signs of congestive heart failure, as illustrated in the case [8]. Most patients have no previous history of heart disease, suggesting that underlying vasculitis is the primary cause [9]. To our knowledge, our case appears to be one of the only cases of myopericarditis in a patient with cryoglobinemic renal failure. The patient underwent an aetiological work-up to rule out secondary infectious, immunological and neoplastic causes. Essential cryoglobulinaemia with type 5 cardio-renal syndrome was selected.

Most of the cases described in the literature concern patients with cryoglobulinaemia secondary to HCV [10]. Indeed, Retamozo et al. reported three cases of severe cardiac involvement [7] and the retrospective study of 165 patients by Terrier et al. found cardiac manifestations (mainly chest pain and congestive heart failure) in seven of them (4%) [11]. Cardiac imaging revealed dilated cardiomyopathy in five patients and hypertrophic cardiomyopathy in one patient. In both studies, including ours, the cardiac manifestations were reversible shortly after initiation of glucocorticoids (GCS) and aggressive immunosuppressive treatment with an anti-CD20 drug [11,12].

Rituximab may be considered as a last resort in situations where the risk of mortality is high due to cardiac involvement or the occurrence of severe infectious complications that prevent the use of conventional immunosuppressive treatments. In the latter case, rituximab is a relatively safe drug with a fairly specific mode of action and a limited side-effect profile [13-15]. In terms of outcome, studies have shown that patients with cardiac lesions have poorer long-term survival, in contrast to the results of our patient, in whom we observed spectacular renal and cardiac progression after rapid initiation of treatment.

Diagnosis of cryoglobulinemic vasculitis, particularly when associated with rare complications like cardiac involvement, poses significant challenges due to its clinical variability, limited knowledge of the underlying mechanisms, the absence of clear diagnostic criteria and often non-specific initial symptoms [16]. Early and accurate diagnosis is crucial for preventing severe outcomes such as heart failure, yet it is often delayed due to multiple factors. The diagnosis hinges on detecting cryoglobulins in the blood, but this test is highly sensitive to pre-analytical factors. Proper sample collection, transportation, and processing are critical, as improper handling can lead to false-negative results [17].Cryoglobulins precipitate in cold conditions, and testing requires strict temperature control, which is difficult to maintain outside specialized settings [18]. Additionally, low levels of cryoglobulins or intermittent circulation can complicate the diagnosis.

The relationship between the cardiac involvement observed and the other clinical manifestations, the cryoglobulinaemia assay and the renal biopsy results (which confirmed cryoglobulinemic vasculitis as the mechanism of injury), as well as the overall response to immunosuppressive treatment, strongly suggests a causal link. Further work is needed to investigate the mechanisms underlying how essential cryoglobulinaemia may affect the myopericardium.

## Conclusions

Cryoglobulinemic vasculitis has a poor prognosis due to its aetiology, multi-organ involvement and the severity of organ damage. Microvascular involvement, which affects less than 5% of patients, can simultaneously affect organs such as the kidneys, heart, lungs and digestive tract. The clinical challenge in these acute forms lies in the rapid onset of multiple organ failure, requiring immediate intervention and potentially delaying specific treatment for the vasculitis. However, in cases of suggestive clinical presentation with severe failure of at least one organ, aggressive treatment should be initiated rapidly without waiting for biological or histological confirmation.

## References

[1] Desbois AC, Cacoub P, Saadoun D (2019). Cryoglobulinemia [Article in French]. Rheumatol Rev.

[2] Terrier B, Marie I, Lacraz A (2015). Non HCV-related infectious cryoglobulinemia vasculitis: results from the French nationwide CryoVas survey and systematic review of the literature. J Autoimmun.

[3] Liu K, Cedres LB, Stamler J (1982). Relationship of education to major risk factors and death from coronary heart disease, cardiovascular diseases and all causes, findings of three Chicago epidemiologic studies. Circulation.

[4] Schultz WM, Kelli HM, Lisko JC (2018). Socioeconomic status and cardiovascular outcomes: challenges and interventions. Circulation.

[5] Borkowski P, Borkowska N, Mangeshkar S, Adal BH, Singh N (2024). Racial and socioeconomic determinants of cardiovascular health: a comprehensive review. Cureus.

[6] Brouet JC, Clauvel JP, Danon F, Klein M, Seligmann M (1974). Biologic and clinical significance of cryoglobulins. A report of 86 cases. Am J Med.

[7] Retamozo S, Díaz-Lagares C, Bosch X (2013). Life-threatening cryoglobulinemic patients with hepatitis C: clinical description and outcome of 279 patients. Medicine (Baltimore).

[8] Terrier B, Krastinova E, Marie I (2012). Management of noninfectious mixed cryoglobulinemia vasculitis: data from 242 cases included in the CryoVas survey. Blood.

[9] Leleux C, Zerbib Y, Pommerolle P, Da Rocha A, Serpier M, Caillard P (2023). Rare manifestations of cryoglobulinemic vasculitis: a case report. Front Immunol.

[10] Terrier B, Karras A, Cluzel P, Collet JP, Sène D, Saadoun D, Cacoub P (2013). Presentation and prognosis of cardiac involvement in hepatitis C virus-related vasculitis. Am J Cardiol.

[11] Terrier B, Saadoun D, Sène D, Scerra S, Musset L, Cacoub P (2010). Presentation and outcome of gastrointestinal involvement in hepatitis C virus-related systemic vasculitis: a case-control study from a single-centre cohort of 163 patients. Gut.

[12] Zaidan M, Mariotte E, Galicier L (2012). Vasculitic emergencies in the intensive care unit: a special focus on cryoglobulinemic vasculitis. Ann Intensive Care.

[13] Ghijsels E, Lerut E, Vanrenterghem Y, Kuypers D (2004). Anti-CD20 monoclonal antibody (rituximab) treatment for hepatitis C-negative therapy-resistant essential mixed cryoglobulinemia with renal and cardiac failure. Am J Kidney Dis.

[14] Barton JC, Herrera GA, Galla JH, Bertoli LF, Work J, Koopman WJ (1987). Acute cryoglobulinemic renal failure after intravenous infusion of gamma globulin. Am J Med.

[15] Campise M, Tarantino A (1999). Glomerulonephritis in mixed cryoglobulinaemia: what treatment?. Nephrol Dial Transplant.

[16] Galli M, Monti G, Marson P (2019). Recommendations for managing the manifestations of severe and life-threatening mixed cryoglobulinemia syndrome. Autoimmun Rev.

[17] Motyckova G, Murali M (2011). Laboratory testing for cryoglobulins. Am J Hematol.

[18] Sargur R, White P, Egner W (2010). Cryoglobulin evaluation: best practice?. Ann Clin Biochem.

